# Reserpine methonitrate, a novel quaternary analogue of reserpine augments urinary excretion of VMA and 5-HIAA without affecting HVA in rats

**DOI:** 10.1186/1471-2210-4-30

**Published:** 2004-11-16

**Authors:** Satyanarayana Sreemantula, Krishna M Boini, Srinivas Nammi

**Affiliations:** 1 Pharmacology Division, Department of Pharmaceutical Sciences, Andhra University, Visakhapatnam 530003, Andhra Pradesh, INDIA; 2 Current address: Department of Physiology, University of Tübingen, D 72076 Tübingen, GERMANY

**Keywords:** Resperine methonitrate (RMN), Resperine, Biogenic amines, Urinary metabolites, Blood pressure, Rats

## Abstract

**Background:**

Reserpine, an alkaloid from *Rauwolfia serpentina *was widely used for its antihypertensive action in the past. In later years, its use has been reduced because of precipitation of depression and extra pyramidal symptoms due to its central action. In the present investigation, reserpine methonitrate (RMN), a novel quaternary analogue of reserpine was synthesised and evaluated biochemically for its central and peripheral amine depleting actions in rats while its influence on the blood pressure was measured in anaesthetized rats in comparison with reserpine

**Results:**

Reserpine treatment (5 mg/kg) produced a significant increase in the urinary excretion of VMA, 5-HIAA and HVA while RMN at doses of equal to and double the equimolar doses of reserpine (5 and 10 mg/kg) produced significant increase in VMA and 5-HIAA excretion without producing any effect on HVA excretion compared to control animals. Reserpine in the dose range of 0.5 to15 μg/kg produced significant reduction in blood pressure compared to control. RMN was also found to produce significant decrease in blood pressure at doses of 10, 25 and 50 μg/kg body weight in comparison to control. The results indicated peripheral depletion of biogenic amines by RMN without affecting the central stores of the amines.

**Conclusions:**

The present study clearly indicated that the quaternization of reserpine restricts its transfer across the blood-brain barrier and could be the reason for its selective peripheral action. It is also clear that the hypotensive actions of RMN could be due to peripheral depletion of catecholamines.

## Background

Reserpine, an alkaloid isolated from *Rauwolfia *species, was introduced for the treatment of hypertension and schizophrenia in 1950's but was replaced by more effective drugs by the end of 1970's [1-6]. Reserpine is known to act centrally as well as peripherally by depletion of biogenic amines like noradrenaline, dopamine and serotonin. Mostly, its peripheral depletion of amines is responsible for its antihypertensive effect while its central depletion of amines is responsible for its antipsychotic action [7-13]. However, because of its central action it produces sedation and Parkinsonism when used for the management of hypertension for prolonged periods [14-17]. As a result it has reduced usage for chronic treatment in hypertensive patients and its use is limited to selective patient population only [18,19]. Hence there is a need for structural modification of the drug to make it more acceptable therapeutically for the treatment of hypertension.

Attempts were made in the past to synthesize derivatives of reserpine with possibly higher and/or modified activities or with fewer side effects [20-23]. Compared to reserpine itself, a number of reserpine analogues were found to exert a stronger influence on the amine concentration in the periphery than in brain [24-26].

Based on the poor ability of quaternary compounds to penetrate the blood-brain barrier, a great deal of research has been devoted towards quaternization of existing drugs to achieve preferential peripheral action [27-32]. Earlier reports have demonstrated the synthesis of quaternary derivatives of reserpine and isoreserpine, however their pharmacology was not studied [33,34]. Previous studies by our group also revealed that reserpine methiodide produced selective depletion of peripheral biogenic amines without affecting their central stores [35]. In the present investigation, a quaternary analogue of reserpine *viz*., reserpine methonitrate (RMN), which was synthesized in our laboratory was evaluated in rats for its amine depleting actions compared to reserpine. For this, the urinary levels of vanillylmandelic acid (VMA), 5-hydroxyindoleacetic acid (5-HIAA) and homovanillic acid (HVA) which are the respective metabolites of noradrenaline, serotonin and dopamine were estimated after reserpine or RMN treatment in rats. The change in the blood pressure response of anaesthetized rats after treatment with RMN was also evaluated in comparison to reserpine.

## Results

### Biochemical estimations

**Figure 1:**
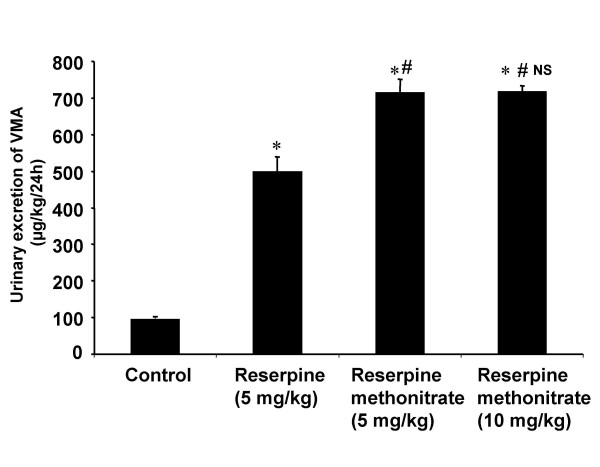
**Diagram illustrating the effect of reserpine and reserpine methonitrate on the 24 h urinary excretion of VMA in rats. **Each bar indicates the mean excretion of six animals. Significant difference from control group: *p < 0.05 Significant difference from reserpine treated group: #p < 0.05 NS-No significant difference between 5 and 10 mg/kg treated groups of reserpine methonitrate

The main aim of the study was to determine whether RMN was able to deplete the central and peipheral biogenic amines to the same extent as produced by reserpine. Reserpine at a dose of 5 mg/kg body weight produced significant increase in the urinary excretion profile of VMA compared to control animals. The analogue at doses equimolar to reserpine of 5 and 10 mg/kg body weight produced more significant increase in VMA excretion compared to controls and that observed with reserpine (Fig 1). However, the higher dose (10 mg/kg body weight) of RMN did not further enhance the excretion of VMA produced by 5 mg/kg body weight dose.

**Figure 2:**
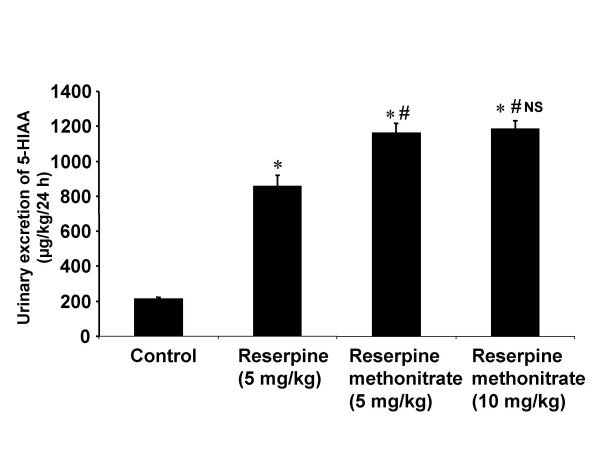
**Diagram illustrating the effect of reserpine and reserpine methonitrate on the 24 h urinary excretion of 5-HIAA in rats. **Each bar indicates the mean excretion of six animals. Significant difference from control group: *p < 0.05 Significant difference from reserpine treated group: # p < 0.05 NS-No significant difference between 5 and 10 mg/kg treated groups of reserpine methonitrate

Significant increase in 5-HIAA excretion was observed with reserpine at a dose of 5 mg/kg body weight and with the equivalent dose of RMN (Fig 2). The amount of 5-HIAA excreted in animals treated with the analogue/reserpine was found to be more than in the control. However the effect was found to be more with analogue compared to reserpine. The enhancement in dose to 10 mg/kg body weight of RMN did not produce any further increase in 5-HIAA excretion.

**Figure 3:**
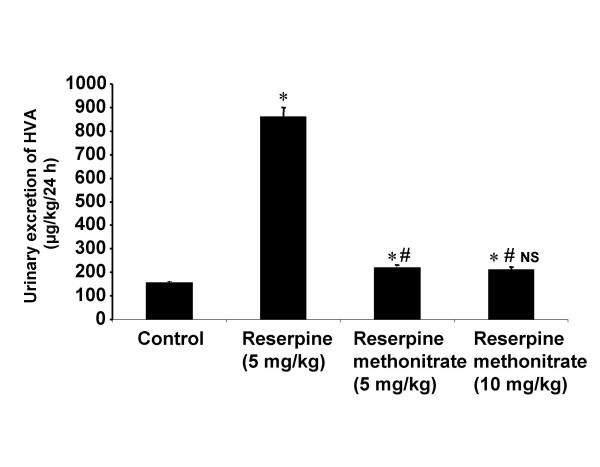
**Diagram illustrating the effect of reserpine and reserpine methonitrate on the 24 h urinary excretion of HVA in rats. **Each bar indicates the mean excretion of six animals. Significant difference from control group: *p < 0.05 Significant difference from reserpine treated group: # p < 0.05 NS-No significant difference between 5 and 10 mg/kg treated groups of reserpine methonitrate

A marked increase in the HVA excretion was observed in animals treated with reserpine compared to controls while minor change was observed in animals treated with RMN at doses of 5 and 10 mg/kg body weight compared to control (Fig 3).

### Effect on normal blood pressure of anaesthetized rats

The effect of reserpine and RMN on the normal blood pressure of anaesthetized rats was shown in Table 1. Dose dependent hypotension was observed with reserpine as well as with RMN. However, the vehicle (DMSO) also produced hypotension which was approximately 15 mm Hg from basal level. Reserpine at doses of 0.5, 1, 5, 10 and 15 μg/kg produced significant (p < 0.01) reduction in blood pressure compared to control. RMN was also found to produce significant (p < 0.01) decrease in blood pressure at doses of 10, 25 and 50 μg/kg body weight compared to control

**Table 1:** Effect of reserpine and reserpine methonitrate on the mean arterial pressure of anaesthetized rats.

**Drug**	**Dose (μg/kg)**	**Mean arterial pressure (mmHg, n = 6)**			**Reduction due to drug**
			
		**Before drug**	**After drug**	**Mean reduction**	
Vehicle	0.05 ml	126.2 ± 2.8	110.0 ± 4.1	16.2 ± 1.4	---
Reserpine	0.25	138.3 ± 4.6	119.5 ± 4.2	18.8 ± 1.4^NS^	2.6
	0.50	134.1 ± 5.5	91.2 ± 5.0	42.9 ± 0.9**	26.5
	1	135.2 ± 4.7	80.5 ± 3.8	54.8 ± 1.6**	38.6
	5	130.5 ± 5.1	69.0 ± 3.5	61.5 ± 2.8**	45.3
	10	130.0 ± 4.2	58.7 ± 3.9	71.2 ± 1.3**	55.0
	15	131.5 ± 5.1	41.1 ± 2.9	90.4 ± 3.1**	74.2
Reserpine methonitrate equivalent to reserpine	10	128.8 ± 6.4	90.0 ± 9.1	38.8 ± 3.6**	22.6
	25	135.0 ± 8.5	73.0 ± 7.4	62.0 ± 2.8**	45.8
	50	136.8 ± 5.3	42.5 ± 3.7	93.0 ± 8.0**	76.8

Significant difference from DMSO treated group: **p < 0.01

NS – No significant difference from DMSO treated group.

## Discussion

The structural modification of existing drugs to achieve selective action is not uncommon in providing better pharmaceutical care to the needy patients. It has been well established that the antihypertensive and tranquilizing actions of reserpine are mediated through the depletion of biogenic amines in the body [12,13,36]. The peripheral depletion of amines is responsible for its antihypertensive effect [11,37] while their central depletion plays a role in sedation and depression of reserpine [38,39]. Reserpine exerts its depleting effect by specifically inhibiting the adenosine triphosphate-Mg^2+^-dependent incorporation of biogenic amines into their storage vesicles [40,41].

Since reserpine depletes noradrenaline, 5-HT and dopamine from their storage sites, this results in a consequent increase in their metabolite levels in urine. Previous investigators have demonstrated a marked increase in the urinary excretion of peripheral and central metabolites of biogenic amines in animals treated with reserpine [8,42-44].

In the present investigation, a non-invasive biochemical approach was followed to determine the 24 h urinary excretion of VMA, 5-HIAA and HVA in rats treated with reserpine or RMN. Moreover, VMA, the peripheral metabolite of noradrenaline; 5-HIAA, the main metabolite of serotonin; and HVA, the predominant metabolite of dopamine were selected as the biomarkers for evaluation since noradrenaline exists both centrally and peripherally, serotonin exists mainly peripherally while majority of dopamine exists centrally. Since 99% of the total body's content of serotonin is present in the periphery, it is considered that the major part of the excreted 5-HIAA is from the peripheral release [45,46]. Similarly, high levels of dopamine are found in the centre rather than periphery, and any change in the HVA excretion in urine was considered as a corresponding change in dopamine levels at the central regions [47]. These indices provide an indirect evidence for the peripheral and central monoamine depleting effects of reserpine and its quaternary analogue.

The results showed that reserpine increased the urinary excretion of VMA, 5-HIAA and HVA indicating the depletion of peripheral as well as central biogenic amines. These are in agreement with the results observed by previous investigators [8,35,42-44]. The increase in the urinary excretion of VMA and 5-HIAA with RMN is higher than with reserpine at equimolar dose of 5 mg/kg body weight. The localized distribution of the analogue in the periphery could led to higher level of depletion of peripheral noradrenaline and serotonin hence their metabolite levels were found to be increased much more which also substantiate our previous studies [35]. The inability of the analogue to increase HVA excretion unlike reserpine could be due to its non-entry across the blood-brain barrier and into the central nervous system to deplete dopamine which is present predominantly in mesolimbic, nigrostriatal and tuberoinfendibular systems [48].

The increased urinary levels of 5-HIAA observed with RMN could be due to the peripheral release of 5-HT as it is found predominantly at the periphery in enterochromaffin cells. The higher dose (10 mg/kg) of RMN did not produce any further increase in the VMA and 5-HIAA excretion compared to lower dose. The possible reason for this effect could be that 5 mg/kg dose was sufficient to deplete the amines completely from the storage sites.

In order to evaluate whether the quaternary analogue of reserpine (RMN) still retains the peripheral blood pressure lowering activity, further experiments were carried out on the blood pressure of anaesthetized rats. Thus far, the results of RMN on the blood pressure response of anaesthetized rats confirmed that the peripheral actions of reserpine molecule are not affected by quaternization. However, in the present study the vehicle (DMSO) also produced minor hypotensive effect on blood pressure of rats when administered alone with the dose used for the administration of the drugs. Earlier workers [49] also reported hypotension with DMSO supporting the present observations. Reserpine produced dose dependent reduction in blood pressure as demonstrated by previous investigators [50,51]. As indicated in earlier reports [9,11,40,52-54] the hypotensive effect of reserpine observed in rats is due to the depletion of catecholamines from the peripheral stores.

The effect of equimolar doses of RMN also indicated hypotension however, with higher doses compared to reserpine. It is further indicated that quaternization of reserpine not only restricted the entry of RMN to central nervous system but also reduced to the target tissue in the periphery. Hence relatively higher doses were required to produce reserpine like effect. Mechanistically, the hypotensive actions of RMN could also be due to peripheral depletion of catecholamines as evident from the positive correlation with the results of previous section on the peripheral depletion of monoamines.

## Conclusion

In conclusion, the present study indicated that the quaternization of reserpine molecule prevents its access into the central nervous system and thereby produces selective peripheral depletion of biogenic amines. Furthermore, the study indicated that quaternization of reserpine had not abolished the hypotensive response but only higher doses were required.

## Methods

### Chemistry

The synthesis of RMN was done as follows: The solution of reserpine (2 g, 3.3 mmols) in dichloromethane (20 ml) was added to methyl iodide (11 ml, 176 mmols) and the resulting mixture was kept for two days in dark. The solid was filtered and washed with a little cold dichloromethane and dried under vacuum at 70°C for 2 h to yield reserpine methiodide (RMI) [33,34]. Then, to a solution of RMI (0.25 gm, 0.67 mmols) in a mixture of dichloromethane (3 ml) and aqueous ethanol (90%, 2 ml) was added a solution of silver nitrate (56 mg, 0.67 mmols) in aqueous ethanol (90%, 2 ml). The reaction mixture was stirred overnight at room temperature. The solution was filtered and washed thoroughly with chloroform : methanol (1:1). The solid obtained after evaporation of the solvent was passed through a silica gel column and eluted with chloroform : methanol (80:20) to yield RMN, m.p. 292–294°C.

### Chemicals used

Reserpine and thiopentone were generous gift samples from Novartis India Limited and Abbott Laboratories, Mumbai respectively. The standard samples of VMA, 5-HIAA, HVA and iso-VMA (internal standard) were purchased from Sigma-Aldrich, St. Louis, USA. All other chemicals used were of HPLC or analytical grade as appropriate.

The solutions of reserpine and RMN under study were prepared in DMSO and the volume of each dose was adjusted to 0.1 ml/100 gm body weight as suggested by Varma et al., [49]. The doses of RMN were calculated on equimolar basis of reserpine.

### Animal experiments

Albino rats of either sex weighing between 100–150 gm (Charkaborty Enterprise, Kolkata) were used in the study. They were acclimatized to the laboratory conditions for at least 10 days prior to the experiment and were provided with standard diet and water *ad libitum *with 12 h light and dark cycle. The animal experiments conducted in this research work were approved by the Institutional Animal Ethics Committee and by the government regulatory body for animal research (Regd. No. 516/01/A/CPCSEA).

#### Biochemical estimations

Animals were divided into 4 groups of six each and were housed individually in metabolic cages. Funnels of suitable size were arranged at the bottom of the metabolic cages for collection of urine. Perforated plastic discs were arranged in the funnels to retain fecal matter. The animals were maintained at room temperature and acclimatized to metabolic cages for few days prior to drug administration.

The treatment given to the groups of animals was as follows:

Group 1: Control animals treated with DMSO intraperitoneally at a dose of 0.1 ml/100 gm body weight.

Group 2: Animals administered intraperitoneally with reserpine at a dose of 5 mg/kg body weight.

Group 3: Animals administered intraperitoneally with RMN at a dose equivalent to 5 mg/kg body weight of reserpine.

Group 4: Animals administered intraperitoneally with RMN at a dose equivalent to 10 mg/kg body weight of reserpine.

In each group, animals were placed individually in metabolic cages after drug administration and were allowed access to water. The 24 h urine samples from the point of drug administration was collected for each animal in a beaker containing 5 ml of 6 *M *HCl arranged at the bottom of the funnel. The volumes of the 24 h urine samples collected in the beakers were noted individually and about 2 ml of urine (mixture) from each animal was taken into sample tubes and centrifuged at 3000 rpm for 10 minutes. The supernatants were transferred into another set of clean and dry tubes and stored at -20°C until analysis by HPLC.

#### Simultaneous HPLC determination of VMA, 5-HIAA and HVA in urine

The procedure described by Wako-chem. Co.,[55] was used for the simultaneous determination of the above metabolites. The urine samples were thawed before analysis. To 0.2 ml of each sample, 0.1 ml of internal standard (iso-VMA, 1000 ηg) and 0.7 ml of mobile phase were added. The solutions were mixed well and filtered through 0.4 μm membrane filter. The filtrate (20 μL) was injected into the column (RP C-18, 250 mm × 4.6 mm I.D; particle size 5 μm; YMC Inc., USA). The mobile phase (filtered through 0.4 μm membrane filter) comprised of 10:90 v/v of acetonitrile and 0.1 *M *KH_2_PO_4 _and the flow rate of the mobile phase was maintained at 0.8 ml/min, which yields a column back pressure of 220–230 kgf/cm^2^. Detection was done by UV absorption at 230 ηm. The range of the detector was set at 0.001 a.u.f.s. The peak area ratios of VMA, 5-HIAA and HVA to that of internal standard were calculated and substituted in the respective regression equations to estimate the amount of the metabolite present in the sample.

#### Effect on normal blood pressure of anaesthetized rats

The procedure described by Noble [56] was followed to evaluate the effect of RMN on normal blood pressure of anaesthetized rats in comparison with reserpine. Groups of rats of six each were anaesthetized with an intraperitoneal injection of thiopentone (40 mg/kg body weight). The femoral vein was cannulated for administration of supplementary doses of anaesthetic (if required) and drug solutions.

Haemodynamic setup was used to record the blood pressure of rats. The blood pressure of each animal was recorded from left common carotid artery connected to a mercury manometer on kymograph paper. The normal blood pressure of rats was recorded after stabilization for 30 minutes. The different doses of reserpine (0.25, 0.50, 1, 5, 10 and 15 μg/kg body weight) or RMN (10, 25 and 50 μg/kg body weight) were studied in separate groups (n = 6) to determine the change in blood pressure response.

### Statistical analysis

Data are expressed as mean ± standard error of means. Statistical analysis was done using one-way analysis of variance (ANOVA). Post-hoc comparisons were done by using Dunnet's *t*-test. In all the cases, p < 0.05 was considered statistically significant.

## Abreviations

RMN: Reserpine methonitrate

VMA: Vanillylmandelic acid

5-HIAA: 5-Hydroxyindoleacetic acid

HVA: Homovanillic acid

DMSO: Dimethyl sulfoxide

## Authors' contributions

SN made significant contribution in designing the studies, conducting the experiments, interpretation of the data, conceptualization of statistical analyses and drafting the final manuscript. KMB assisted in experimental work, data analysis and writing of the manuscript. SS conceived the study, made substantial contributions in data analysis, data interpretation, writing of the manuscript and in coordination of the experiments. All authors read and approved the final manuscript.
